# Following Randolph Blake's furrow further

**DOI:** 10.1167/jov.25.6.9

**Published:** 2025-05-23

**Authors:** Anna Riga, Stuart Anstis, Ian M. Thornton, Patrick Cavanagh

**Affiliations:** 1 Department of Cognitive Science, University of Malta, Msida, Malta; 2 Department of Psychology, University of California San Diego, La Jolla, CA, USA; 3 Department of Psychology, Glendon College, North York, ON, Canada; 4 CVR, York University, North York, ON, Canada; 5 Psychological and Brain Sciences, Dartmouth College, Hanover, NH, USA

**Keywords:** motion, position, motion-induced position shift

## Abstract

In 1992, Randolph Blake, in collaboration with Robert Cormack and Eric Hiris, reported a strong deviation in perceived direction for a target moving over an oblique, static grating. Here we follow up on this effect, subsequently called the *furrow illusion*, to determine its origin. We find, unlike Cormack et al., that it is influenced by the luminance of the target and that it does not survive smooth pursuit of a moving fixation that stabilizes the target on the retina. We also introduce an inverted version of the furrow stimulus with the static grating visible only within the moving target rather than only around it. This “peep-hole” furrow stimulus shows a similar deviation in its direction and is quite similar to the well-known double-drift stimulus (Lisi & Cavanagh, 2015). Like the double-drift but unlike the furrow stimulus, its illusory direction persists when tracking a fixation that moves in tandem with the target. The main source for the illusion in both cases appears to be the terminators where the grating's bars meet the target contour. These terminators move laterally along the target's contour as the target moves vertically and the combination of these two directions creates the illusory oblique motion. However, the loss of the illusion for the tracked furrow stimulus suggests either a contribution from negative afterimages within the target or from induced motion in this case.

## Introduction

Randolph Blake has had an extraordinarily creative and varied career. His work on binocular rivalry is certainly his most well-known research interest, and many of the articles in this special issue build on his work in this area. But he has made contributions across many other areas. Here we have picked up one of his discoveries in the field of motion and explored the mechanisms underlying the effects he reported.

Thirty years ago, Randolph Blake phoned one of us (SA) in a state of great excitement to announce that he had discovered a new illusion of motion. Working with his colleagues Cormack and Hiris, he had noticed that when a short line moved across a large oblique grating and (importantly) was viewed in peripheral vision, then its perceived direction of motion was often at variance with its true direction (Cormack, Blake, & Hiris, 1992, see Movie 1). Randolph asked Stuart why he thought it happened. Stuart mumbled something about intersections, an explanation that had already occurred to Randolph, and Stuart thought no more about it.

**Movie 1.** The first report (Cormack, Hiris, & Blake, 1992). A short horizontal line moves vertically up and down across a field of oblique static 45° stripes. Use this link to open the movie in a browser window: https://cavlab.net/Demos/Furrow/#0/. When viewed in peripheral vision, the line appears to move obliquely, almost along the grating's bars. In contrast, a vertical line moving up and down is seen veridically. These results can be explained by the motions of the intersections between the static 45° stripes and the moving bars. Intersections with the vertical bar do not move at all. Whereas those with a horizontal bar move down to the left, along the stripes, when the bar moves down, and up to the right when the bar moves up. Both of these pull the horizontal bar's motion onto an oblique path. Adapted from Cormack et al., 1992.

Twenty years later Stuart discovered an exciting new phenomenon that he dubbed the *furrow illusion*. Only after he had published it in the *Journal of Vision* (Anstis, 2012) did he realize that he had merely rediscovered and renamed Randolph Blake's original discovery. So, Stuart, together with Patrick Cavanagh published another article (Anstis & Cavanagh, 2018) including, at last, appropriate credit to the Cormack, Blake, and Hiris paper. Remy Allard and Jocelyn Faubert (2016) have also published further studies on the furrow illusion as have Shapiro, Knight, and Lu (2010), although they called it the *escalator illusion*.

**Figure 1.**
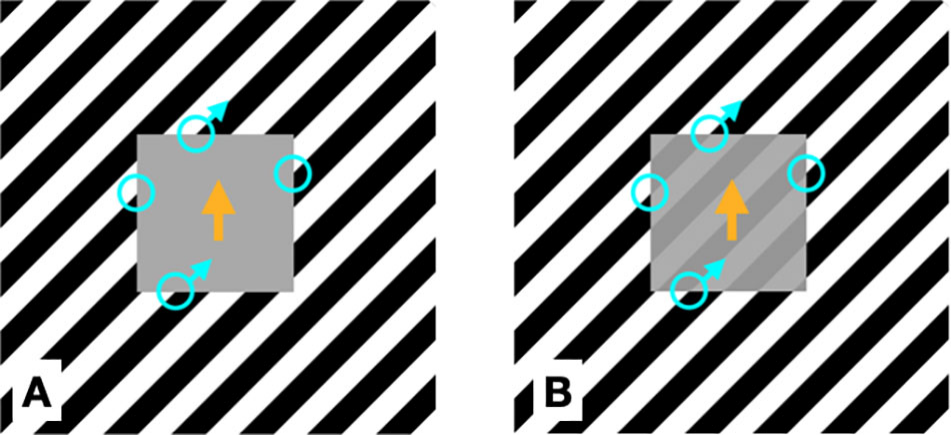
(**A**) As the square moves up, the intersections slide up and to the right on the top and bottom but on the sides the intersections remain fixed in place relative to the screen. The oblique motion of the intersections along the top and bottom edges adds to the vertical motion to make the square appear to drift up to the right (Cormack et al., 1992; Anstis, 2012). (**B**) Because the background grating is static, a negative afterimage will build up during steady fixation and be visible inside the gray square. This afterimage may add to the strength of the edge intersections (Thornton & Riga, 2024).

The previous articles reported several interesting features of the furrow illusion*.* There is no illusion at the fovea and the illusion strength increases fairly linearly with eccentricity (Cormack et al., 1992; Anstis, 2012; Allard & Faubert, 2016). Cormack et al. (1992) and Anstis (2012) showed that the presence of the illusion in the periphery but not the fovea cannot be attributed to blurring in the periphery as a blurred stimulus still did not produce an illusion in the fovea. Cormack et al. (1992) also reported that the illusion was undiminished if the moving bar and the grating were in different depth planes. Anstis and Cavanagh (2018) showed that when a square moved vertically up and down over an oblique grating, it was the leading and trailing edges that produced the change in perceived path, not the sides. This is consistent with the role of the edge intersections in producing the effect (Figure 1) as originally suggested by Cormack et al. (1992). Some results suggest a rather high level of processing before the emergence of the illusion. In particular, Cormack et al. (1992) had participants track a fixation point that moved in tandem with the moving bar, keeping the bar more or less fixed on the retina. Despite the absence of retinal motion signals for the bar, the strength of the illusion was unaffected (although see Experiment 3 here). Anstis and Cavanagh (2018) had a square target move up and down within a narrow vertical column of an oblique grating that was flanked with similar narrow gratings alternating between left tilt and right tilt. As the square moved up and down in one of the columns, an oblique path was seen that was consistent with the grating's orientation in that single column. This was surprising because observers could not report the orientation of the column where the square was located. The columns were close enough together and far enough in the periphery to be crowded (e.g., Levi, 2008) so there was no access to the individual orientations within each column, but the furrow effect was undiminished. Importantly, the moving square appeared to travel across several columns even though physically it remained within the single, original one. These results and similar ones from Allard and Faubert (2016) argue that the effect does not come from tracking the individual edge features but from low-level motion responses to these features which produce an oblique motion vector that is used at a higher level to construct a perceived path.

The furrow stimulus shares many features with the double-drift illusion (Lisi & Cavanagh, 2015; also known as the Infinite Regress illusion, Tse & Hsieh, 2006, and the curveball illusion, Shapiro et al., 2010). Movie 2 shows the furrow stimulus on the left, the double-drift in the center, and what we call the *peep-hole furrow* on the right. In the peep-hole furrow, the static grating is visible only through the moving target disk and is surrounded everywhere else by uniform gray background.

**Movie 2A.** (**A**) The furrow illusion (Anstis, 2012). The gray disk moves up and down over a static, oblique grating but when viewed in the periphery, its path is tilted to the right. Click here to open the movies in your browser window and resize them so the target is at least 1 dva. (**B**) The double-drift illusion (Lisi & Cavanagh, 2015).

**Movie 2B.** (**B**) The double-drift illusion (Lisi & Cavanagh, 2015). The disk moves up and down and an internal texture moves left and right. The two motions reverse at the same time so that the internal motion drags the disk to the right on the way up and drags it to the left on the way down, both generating a rightward tilt in the path.

**Movie 2C.** (**C**) The peep-hole furrow illusion. Here the disk of the furrow stimulus becomes a hole through which we see the grid that is now behind the gray background. It is the exact reverse of the furrow stimulus. All three stimuli generate similar oblique motion paths when viewed in the periphery. Go here to open the movies in a browser window: https://cavlab.net/Demos/Furrow/#2/.

Thornton and Riga (2024) used a variant of the furrow illusion to examine the impact of negative afterimages on peripherally viewed objects. The steady fixation usually adopted when viewing displays with static grating backgrounds quickly leads to a build-up of afterimages. As a target moves across the background grating, these afterimages are visible within the untextured target, being most prominent at the leading edge. Thornton and Riga (2024) suggested that when viewed peripherally, such afterimages could interact with the target contours. In addition to causing shape distortions—the so-called “partial object doubling” described in their article—such interactions could also potentially modulate the furrow illusion itself. For example, afterimages could help sharpen the contrast of the edge intersections thought to provide local oblique motion signals (Cormack et al., 1992; Anstis, 2012). Anstis (2012) also introduced a “negative lens” version where the moving disk was also filled with the background grating but with reversed contrast. As Thornton and Riga (2024) pointed out, the negative lens stimulus mimics the naturally occurring afterimages within the original blank target, except they are higher in contrast and there is no gradient of strength across the target. The negative lens stimuli and the novel “peep-hole furrow” stimuli introduced in Movie 2 both have a physical internal texture that generates a motion vector relative to the target (the texture moves relative to the target even though static relative to the display). It is as yet unclear whether the internal afterimages contribute to the illusory motion in the furrow stimulus.

In this article, we first examine the role of the target's luminance. Cormack et al. (1992) claimed that there was no effect of changing the target's luminance from white through gray to black in the furrow effect. In contrast, the double-drift illusion is strongest when the mean luminance of the drifting target matches that of the gray surround and weak or absent when the target luminance has no overlap with the background luminance (Gurnsey & Biard, 2012). The second experiment compares the illusion strength for the furrow (Movie 2A) and its complement, the “peep-hole” furrow stimulus (Movie 2C).

Our third experiment compares the illusion strength for the furrow and peep-hole furrow stimuli while observers are tracking a fixation point that moves in tandem with the target. This effectively nulls the motion of the target on the retina. The double-drift stimulus has been shown to produce a strong illusion even when smooth pursuit has eliminated the retinal motion of the target (Cavanagh & Tse, 2019).

## Experiment 1: Target luminance

This experiment examines whether the illusion is influenced by the target's luminance.

### Participants

The twelve participants (seven females; age range 18-32, mean 23; one left-handed) all had normal or corrected-to-normal vision and were naive as to the purpose of the study. Informed consent was obtained from each before the experimental session. All procedures conformed to the research ethics and data protection guidelines of the University of Malta https://www.um.edu.mt/research/ethics/researchethicsatum/.

### Equipment

The stimuli and task were developed using custom HTML and JavaScript routines. These are available on the OSF page associated with this article at https://osf.io/k7mva/. Stimuli were presented on an Apple iMac 21.5-inch display (Model number: A1418; Apple, Inc., Cupertino, CA, USA) running macOS Catalina (10.15)*.* The screen resolution was 1920 × 1080 pixels, and the animation frame rate was 60 Hz. Responses were recorded via a standard external USB keyboard. Participants were seated at a distance of 105 cm with their head stabilized via a chin rest. The experimental space was a dimly lit, curtained area within a quiet laboratory.

### Display

On each trial participants were presented with a display such as that shown in Movie 3. The display consisted of a static black-and-white zig-zag grid together with a moving, opaque target that oscillated vertically at the center of the grid. The black and white square wave grid subtended 9.4° × 7° degrees of visual angle (dva) and was positioned in the middle of the monitor display, which had a uniform gray background. The grid was divided into seven equal columns. The lines within the central column, which always contained the target, were constrained to an angle of ±45°. The three columns on either side of the target contained uniform lines that alternated between a CW or CCW orientation relative to the target column, with the precise angle varying randomly between ±20° and ±70°. The spatial frequency of the grid within each column was approximately 2.4 cycles/dva.

**Movie 3.** Furrow test stimulus. The gray disk moves up and down over a central column of a static, oblique grating. Use this link, https://cavlab.net/Demos/Furrow/#8/, to open the movie in a browser window. The central column is flanked by adjacent columns that alternate in orientation to prevent the observer from noticing the orientation of the grid underlying the moving target. The fixation has a marker bar that can be adjusted to match the perceived path angle of the target.

A white fixation cross remained visible throughout the trial and was positioned 8 dva either to the left or right of the central target along the horizontal midline of the display. The target stimulus was an opaque disk with a diameter of 0.93 dva, whose contrast varied randomly across five luminance levels from black to white in equally spaced steps. The target moved smoothly up and down at a constant speed of 11.0 dva/s covering a 10 dva vertical path centered on the display midline. After two oscillatory cycles, a probe bar appeared centered on the fixation cross, with its initial orientation set randomly between −90° and 90°.

### Task

Participants were asked to remain fixated on the white cross for the whole duration of each trial. Their task was to estimate the perceived angle of the target's trajectory by adjusting the matching probe bar when it appeared. The Z and X keys on the keyboard were used for large (5°) adjustment steps and the left and right arrow for smaller (1°) adjustment steps. The probe bar was initially set to a random orientation, and participants had no time constraints for responding. When they were satisfied with their adjustment, they pressed the space bar to move to the next trial. Participants completed two blocks of 40 trials, with a short break in between. The task for Experiment 1 is also available on the OSF page associated with this article at https://osf.io/k7mva/.

### Procedure

After signing the consent form, participants were moved to the experimental space and were again given the instructions orally. Before data collection, they completed practice trials until they were comfortable performing the task. During the main experimental session, participants were alone in the experimental space.

### Design and data analysis

Each participant completed two blocks of 40 trials (2 fixation positions × 2 grid orientations × 5 target luminance levels × 2 repetitions). For the analysis, data were collapsed over the two levels of fixation position and grid orientation. A one-way repeated measures analysis of variance was used to examine the five different levels of the target luminance. Since there was a violation of the sphericity assumption, the Greenhouse-Geisser correction was applied. For post hoc analysis Holm's correction was applied.

### Results

**Figure 2.**
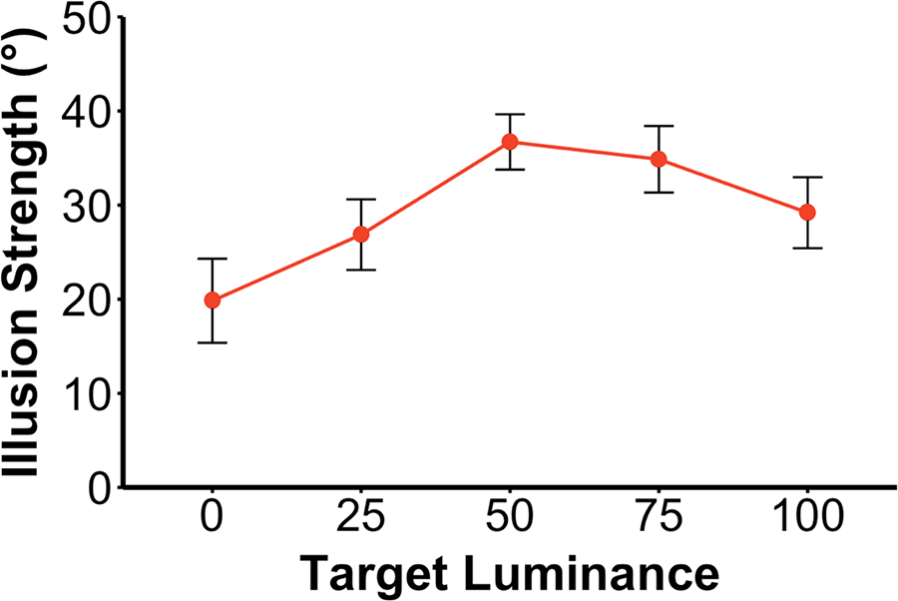
Illusion strength as a function of target luminance. To indicate the illusion strength, the 12 participants adjusted the orientation of a marker bar to match the perceived path. Luminance is given in HSL units of L. The illusion was strongest for 50% (mid-gray) targets and weakest for 0% (black) targets. Vertical error bars show ±1.0 SE.

Figure 2 depicts illusion strength as function of target luminance collapsed across grid orientation. It is clear that the illusion was present across all levels, with a peak at 50% contrast (mid-gray). Analysis indicated a significant effect of target contrast, *F*(1.88, 20.64) = 13.52, *p* < 0.001, $\eta_{p}^{2}$ = 0.56. Post hoc pairwise comparisons showed that the illusion strength for the 0 luminance (black) target was significantly less than for any other target. The only other significant difference was for the 25% target (dark gray) which gave rise to a smaller illusion than 50% target (mid-gray; see OSF Table 1 for details at https://osf.io/k7mva/).

### Discussion

In opposition to Cormack et al.’s claim, the target luminance significantly affected the illusion strength, and was strongest for the mid-gray targets. The increase for the mid-gray target is likely explained by the contrast between the target and the background that follows a U-shape function as the target luminance increases. Specifically, the target is on a striped background so its contrast with the background (averaged along its contour) is lowest when the target is mid-gray (50%) and highest when it is white or black. Pattern vision can resolve the bars of the striped background in the periphery but the motion-selective units have larger receptive fields (e.g., Churchland, Priebe, & Lisberger, 2005), leading them to average over the light and dark bars. In other words, at 50% target luminance, the target is equiluminous with the average luminance of the background, making it a second-order stimulus, a factor that reduces its motion signal. After Cormack et al. (1992) and subsequent articles (Anstis, 2012; Allard & Faubert, 2016; Anstis & Cavanagh, 2018), the illusory oblique direction is assumed to arise from the combination of the target's vertical motion with the lateral motion of the grating's terminators that run along the target's contour (Figure 1). When the target is equiluminous with the average background luminance, the reduced motion from the target itself then gives the secondary, lateral motion from the intersections a relatively larger contribution, increasing the degree of tilt in the perceive motion. As the difference between the target luminance and the average background increases in either direction, the target's vertical motion signal increases, reducing the tilt of the perceived direction. Nevertheless, the illusion remained significant when the target matched either the black bars or the white bars, although it was definitely stronger when white. It appears that the furrow illusion is more robust than the double-drift in the face of a mismatch between the mean luminance of the target and the background.

## Experiment 2: Furrow versus peep-hole furrow

Here we compare the illusory direction seen in the furrow illusion (Movie 2A) to that seen in its complement, the peep-hole furrow, where a hole in a gray surface runs over the static, oblique grating (Movie 2C). This second stimulus is related to the double-drift stimulus (Movie 2B) but has a static oblique texture inside the aperture rather than a grating moving orthogonally to the aperture. Its edge features closely match those of the furrow stimulus except that the intersections of the grid and the target contour are inside the target rather than outside. We asked the participants to report the strength of the illusion by adjusting the orientation of a bar to match the perceived direction of the moving disk.

### Methods

#### Participants

Twelve individuals participated in Experiment 2 (six females; age range 18-33, mean 22; one left-handed). All participants had normal or corrected-to-normal vision, and all were naive as to the purpose of this study. Informed consent was obtained from each participant before the experimental session. All procedures conformed to the research ethics and data protection guidelines of the University of Malta.

#### Equipment and procedure

The equipment and procedure were the same as in Experiment 1. The task for Experiment 2 is also available on the OSF page associated with this article at https://osf.io/k7mva/.

#### Display

Experiment 2 contained two basic displays. The “furrow” display was identical to that described in Experiment 1 with the contrast of the opaque target always set to the same 50% contrast (mid-gray) as the wider background. The second “peep-hole” display varied in two important respects. First, the central grid column was occluded with a gray masking rectangle, so that the underlying ±45° square wave grid was not visible. Second, rather than an opaque target, an aperture was cut in the gray masking rectangle, revealing the grid below (Movie 4). Both target types had the same diameter (0.93 dva) and oscillated vertically at 10.8 dva/s until a response was made. After two oscillatory cycles, a probe bar appeared at the same position as the fixation cross, with its initial orientation set randomly between −90° and 90°.

**Movie 4.** Experiment 2, peep-hole furrow test stimulus. The central column is inverted compared to the furrow stimulus (Movie 3). To open the movie in a browser window, use this link, https://cavlab.net/Demos/Furrow/#10. The static grid is now visible only through the target disk and the column is the background gray elsewhere. The inverted column is again flanked by adjacent columns that alternate in orientation. The fixation has a marker bar that can be adjusted to match the perceived path angle of the target.

#### Task

The task was identical to the one in Experiment 1. Participants were asked to remain fixated on the white cross for the whole duration of each trial. Their task was to estimate the perceived orientation of the target's trajectory by matching it with the probe bar when it appeared. Participants completed two blocks of 32 trials, with a short break in between.

#### Design and data analysis

Each participant completed two blocks of 32 trials (2 fixation positions × 2 grid orientations × 2 target types × 4 repetitions). For the analysis, data were collapsed across fixation position and grid orientation. A paired-samples *t*-test was used to directly compare performance between the furrow and the peep-hole displays.

### Results

**Figure 3.**
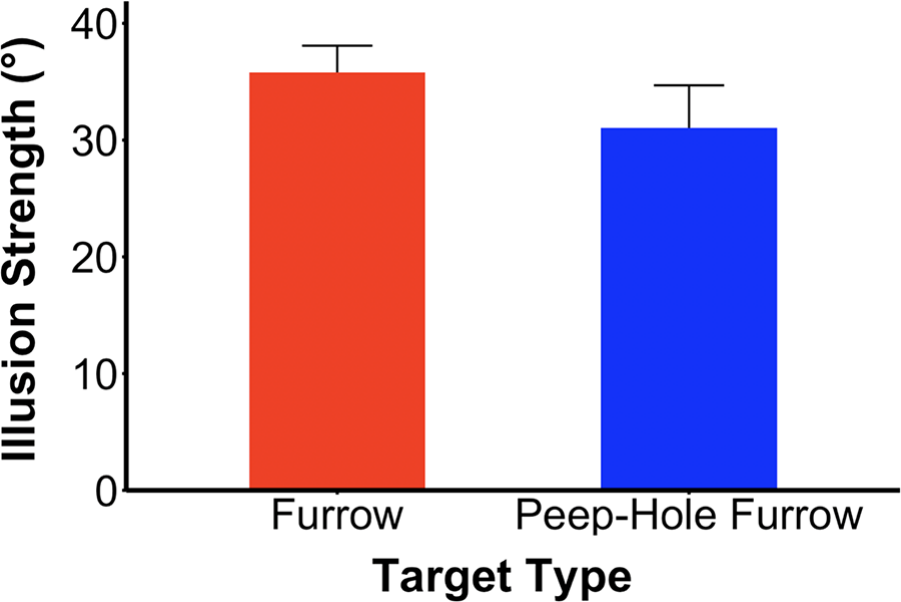
Illusion strength for furrow vs peep-hole furrow. To indicate the illusion strength, the 12 participants adjusted the orientation of a marker bar to match the perceived path. With static fixation, both the furrow and peep-hole furrow show a strong illusion, replicating Experiment 2. The peep-hole furrow was also strong but smaller than the regular furrow effect. Vertical error bars show +1.0 SE.

Figure 3 depicts illusion strength for the furrow and peep-hole targets. The settings for the furrow display (*M* = 35.8° ± 2.3°) replicate those seen in Experiment 1 for middle gray targets, with a clear illusory deviation. The peep-hole display also gave rise to a clear illusion (*M* = 31.1° ± 3.6°), although consistently smaller than that for the furrow, *t*(11) = 2.34, *p* = 0.04, Cohen's *d* = 0.68.

### Discussion

The illusion sizes for both the peep-hole furrow and furrow were quite large, with the peep-hole version at 87% of the magnitude for the furrow version. Assuming that the motion that causes the deviation from the veridical path arises from the intersections, this result shows that the intersections moving along the target contour can be outside (furrow) or inside (peep-hole furrow) the target. The comparison to the double-drift stimulus is revealing here (Movie 2). In the double-drift, the texture within the target is moving relative to the display, and this motion can drive standard direction selective units that respond to retinal motion. In the peep-hole furrow, there is no retinal motion from the grating visible through the aperture—it is static relative to the display. However, the intersections between the grating and the target contour are not static. Like those of the furrow target, the intersections for the peep-hole furrow do move across the retina. Nevertheless, these motions of the T-junction endpoints should not belong to the target in either furrow or peep-hole furrow version; they are extrinsic junctions (Shimojo, Silverman, & Nakayama, 1989), and their motion is an accident of the occlusion of the grating by the moving disk or aperture. Despite this, these intersection motions do contribute to the perceived direction of the targets, at least in the periphery (Cormack et al. 1992; Anstis 2012) where the occlusion cues are possibly no longer effective in parsing motion sources.

## Experiment 3: Tracking the targets

Here, we add a fixation spot that moves up and down in tandem with the target disk for both the furrow and peep-hole furrow stimuli. With accurate tracking, the target will remain relatively fixed on the retina so that the target's vertical motion vector has to be recovered from the eye movement signals. Will the motions of the intersections along the target's contour still combine with this recovered vertical motion? In a similar experiment, the double-drift illusion was found to be undiminished by smooth pursuit that held the target relatively fixed on the retina (Cavanagh & Tse, 2019). Cormack et al. (1992) also tested the furrow illusion with a fixation that moved in tandem with the target. They claimed, without reporting data, that the furrow illusion was undiminished when the target was held steady on the retina. Finally, because the fixation spot is moving, the oblique grating in the furrow stimulus no longer builds up an afterimage, and no reverse contrast stripes will be seen within the gray disk. This will allow some measure of the contribution of the afterimage.

### Methods

#### Participants

Four experienced observers participated in Experiment 3, two of whom are the authors (three females; age range 22-61, mean: 43). All participants were right-handed and had normal or corrected-to-normal vision. Informed consent was obtained from each participant prior to the experimental session. All procedures conformed to the research ethics and data protection guidelines of the University of Malta.

#### Equipment and procedure

Stimuli were presented on an Apple MacBook Air 13.6-inch display (Model number: MC7U4GR/A) running macOS Sonoma (14.6)*.* The screen resolution was 2560 × 1664 pixels, and the animation frame rate was 60 Hz. Responses were recorded via the keyboard. Participants were seated at a fixed distance of 56 cm. The task for Experiment 3 is available on the OSF page associated with this article at https://osf.io/k7mva/. The same procedure as in the previous experiments was followed except that the fixation point could be either static or move in tandem with the target.

#### Display

The furrow display consisted of a static, oblique, black and white grid together with a moving, opaque target that oscillated vertically at its center (Movies 5A and C). The black and white square wave grid subtended 9 × 9 dva and was positioned in the middle of the monitor display, which had a uniform gray background. The lines within the grid had an angle of ±45° and the spatial frequency was 1.7 cycles/dva. The “peep-hole” display was identical to the one in Experiment 2, except that it was not surrounded by grid-line columns (see Movies 5B and D). Both target types had the same diameter (1.4 dva). They oscillated vertically at 13.0 dva/s, covering a 12 dva vertical path centered on the display midline, until a response was made. A white fixation cross and a thin white line remained visible throughout the trial and were positioned 11 dva either to the left or right of the central target along the horizontal midline of the display. On half of the trials, the fixation cross remained stationary, as in the previous experiments. In the remaining trials, the vertical center of the fixation point was linked to the center of the target. It thus moved up and down at the same speed as the target following the faint parallel white line shown in Movie 5. After two oscillatory cycles, a probe bar appeared at the same position as the fixation cross, with its initial orientation set randomly at either 45° or −45°.

**Movie 5A.** Furrow (left) and peep-hole furrow (right) displays with static or moving fixation. To indicate the illusion strength, the four participants adjusted the orientation of a marker to one of three settings −45°, 0°, or +45°. The fixation and marker bar were either static at the vertical midpoint of the display and faint track line, or they moved up and down in tandem with the target. To open the movies in a browser window, use this link, https://cavlab.net/Demos/Furrow/#12.

**Movie 5B.** 

**Movie 5C.** 

**Movie 5D.** 

#### Task

Participants were asked to fixate the white cross for the whole duration of each trial, regardless of whether it was static or moving. Their task was to indicate whether the target appeared to move vertically or with an oblique, illusory motion path. To indicate their response, they could adjust the probe bar by pressing the right and left arrow keys on the keyboard. Each adjustment step was discrete in steps of 45°. They were told to select 0° if the path appeared consistently vertical or the corresponding 45° angular direction if the path appeared consistently tilted in that direction. The oblique response was not intended to capture the precise illusion strength, but to evaluate the frequency of seeing the illusion tilt. When they were satisfied with their choice, they pressed the space bar to move to the next trial. Participants completed 64 trials.

#### Design and data analysis

Each participant completed 64 trials (2 fixation positions × 2 grid orientations × 2 target types × 2 fixation types × 4 repetitions). For the analysis, frequency data were pooled for all participants and collapsed across fixation position and grid orientation. Chi-square tests of independence were conducted to examine the relationship between target type and fixation type.

### Results

**Figure 4.**
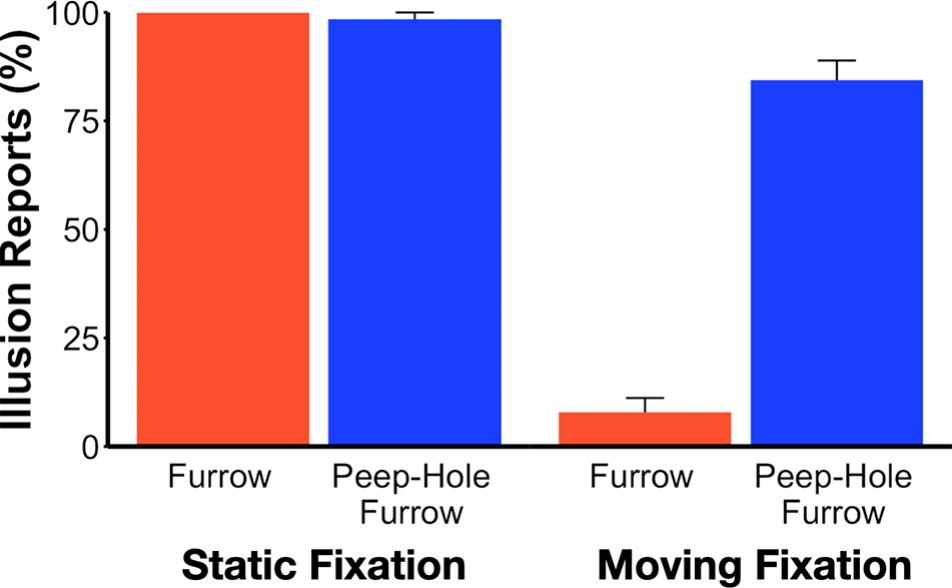
Frequency of reporting illusion direction for static vs moving fixation. With static fixation, the illusion was reported on almost every trial for both the furrow (red bars) and peep-hole furrow (blue bars), replicating Experiment 2. With moving fixation, the peep-hole furrow was again frequently reported, but the furrow effect was virtually eliminated. Vertical bars show +1.0 SE of the mean, n = 4.

Figure 4 provides a summary of the proportion of illusion responses as a function of target type and fixation type, collapsed across the four participants. With static fixation, there was no difference between the two types of display, with participants again reporting an illusion for both the furrow (100%) and the peep-hole furrow (98%), χ² = 0.008, *p* = 0.92, *V* = 0.008. However, with moving fixation, participants almost never reported an illusion for the furrow (8%), although they systematically reported the illusion for the peep-hole furrow (84%), χ² = 40.70, *p* < 0.001, *V* = 0.83. This pattern of results was present in all four participants (see Table 2 available at https://osf.io/k7mva/).

### Discussion

The static fixation condition elicited ceiling level responses in the illusion direction for both the furrow (red) and peep-hole furrow (blue) stimuli. However, the outcome for the moving fixation is quite different. First, the participants still strongly reported the illusion direction for the peep-hole furrow with the moving fixation. This outcome is similar to that for the double-drift stimulus (Cavanagh & Tse, 2019), which also survives smooth pursuit where the target is relatively stationary on the retina. This result requires that the two motion signals, one from the target and one from the intersections, be combined at a fairly high level, after the target's motion is recovered from the eye movement signals. In contrast, for the furrow stimulus, the illusion virtually vanished.

The loss of illusion with smooth pursuit is highly dependent on accurate tracking of the fixation point. At the start of motion or at the turnarounds, this tracking may be lost, and some illusion may be seen. However, observers were asked to judge if the path was *consistently* vertical or oblique rather than if there were some momentary appearance of the illusion (perhaps because of tracking issues).

We considered three possible explanations for this loss of the illusion. First, the target is relatively stabilized on the retina during smooth pursuit, and, although the intersections with the grating bars still slide over the target's contours as they do with static fixation, now there is no retinal motion of the target itself. The target's motion has to be recovered from the eye movement signals and combined with the intersection motion, but perhaps this higher-level combination is blocked or not possible. We have to reject this alternative because it is the same situation for the peep-hole furrow with the exception that the intersections are inside the target rather than outside. This high-level combination was also in evidence for the double-drift stimulus when stabilized during smooth pursuit (Cavanagh & Tse, 2019).

Second, it could be the case that the static background grating is not seen as completely static during pursuit (Filehne, 1922; Mack & Hermann, 1973). The gain of correction for pursuit is typically about 0.8 (e.g., Freeman, 1999) meaning that the background grating is seen to drift backward against the pursuit (and target) direction by about 20% of the target's speed. This residual background grating motion will induce the opposite motion in the target in the direction perpendicular to its oblique orientation (Duncker, 1929). That induced motion is in the direction opposite to the direction of the intersection motion, so perhaps these two cancel. The peep-hole furrow does not have a background grating and so does not face this induced motion, allowing it to retain its full strength.

Finally, because the background grating of the furrow stimulus is moving over the retina during pursuit, it will no longer create negative afterimages within the target. If these negative afterimages are important for the generation of the illusion, then it will be reduced during pursuit. We do not have any result here that can distinguish between these last two alternatives.

## General discussion

We first examined the influence of the target luminance on the furrow effect. Often illusions maximize the positional uncertainty of the target to boost the effects of a secondary signal. For example, the illusory direction seen in the double-drift stimulus is greatest when the mean target luminance matches the background luminance (Gurnsey & Biard, 2012). Cormack et al. (1992) reported that the furrow illusion does not have that property: the illusion strength was unaffected when they switched from a white to gray to black target. This result was reported without describing the method or presenting the data, so we felt it was important to verify their claim, especially because it was not consistent with the effect of mean luminance seen for the double-drift stimulus. Indeed, in contrast to the finding of Cormack et al. (1992), we did find a strong modulation of the illusion strength by target luminance with the maximum occurring with a mid-gray target. This result is more in line with that seen for the double-drift stimulus and suggests that a high level of positional uncertainty for the target (when it is matched in luminance to the average of the background) maximizes the relative contribution of the secondary motion signal (the lateral motion of the intersections).

Our second experiment introduced a novel stimulus, the peep-hole furrow, that is the complement of the furrow stimulus. The stationary oblique grid is now seen through the target disk, which is surrounded by the uniform gray background, rather than the reverse. The illusion strength was measured for both, and although there was some reduction for the peep-hole display, the overall magnitude was found to be quite similar. In both cases, the target motion drives the intersections between the grid and the target along identical paths with the difference that the intersections are inside the target for the peep-hole furrow but outside it for the furrow. The similarity of illusion strength for the two argues in favor of the role of the intersections. The peep-hole furrow is quite similar to the double drift stimulus, except that the internal texture is static with respect to the screen and the retina (compare Movies 2B and 2C). The explanation of both may lie in the motion signals that the grid terminators generate as they slide along the target contour.

The third experiment examined whether the illusion persisted when the fixation point moved in tandem with the target, reducing or eliminating the motion of the target on the retina. The peep-hole furrow was largely unaffected by the smooth pursuit whereas the effect of the furrow stimulus was strongly suppressed. The persistence of the peep-hole furrow effect, like that of the double-drift (Cavanagh & Tse, 2019) indicates that the motion signal from the target and the motion of the intersections are combined at a high level, after the target's motion is recovered from the eye movement signals. The absence of an illusion for the furrow stimulus contradicts the claim of Cormack et al. (1992) that the furrow effect was undiminished by tracking. They presented no methods or analysis so it is possible that they did not get good tracking from the participants who reported the result. In our experiment here, the illusion was lost during tracking. One possible explanation is that the background appears to move in the direction opposite to the tracking indicating an incomplete compensation for the retinal motion of the background due to the eye movement (Filehne, 1922; Mack & Hermann, 1973). If the compensation were accurate, the background would appear stable but it typically appears to drift at about 20% of the target's speed in the direction opposite to the target's motion (e.g., Freeman, 1999). This apparent drift of the background could create an induced motion of the target itself in the oblique direction perpendicular to the bars of the grid. This would act against the oblique motion signal from the intersection motion and cancel the illusion. Although plausible, this explanation remains to be tested.

Randolph Blake and his co-authors (Cormack et al., 1992) suggested that the furrow illusion was caused by the motion of the grid's intersections with the target. Even though the grid itself was stationary, these intersections ran along the target's contours generating a secondary motion signal. Subsequent articles have agreed that these intersections are the source of the illusion (Anstis, 2012; Allard & Faubert, 2016; Anstis & Cavanagh, 2018). We have continued this support here and extended the stimuli capable of producing this dual motion effect to the peep-hole version where the intersections are inside the target rather than outside. In fact, the intersections may be both inside and outside if we consider the negative afterimages (Thornton & Riga, 2024) or the negative lens version of the furrow stimulus (Anstis, 2012).

The intersections where the grid lines terminate on the target contour would stimulate “end-stopped” cells (Hubel & Wiesel, 1965) that have an inhibitory zone at one end of their oriented receptive fields. These cells respond to oriented contours as long as the contour does not extend into their inhibitory zone. Pack, Livingstone, Duffy, and Born (2003) demonstrated that many end-stopped V1 neurons responded to the motion direction of the contour endpoint independently of the contour's orientation. In MT, Pack, Gartland, and Born (2004) found that cells were more strongly influenced by motion signals from terminators, more so than by the ambiguous signals from the contours themselves, although this dominance took some time (60 ms) to appear (Pack & Born, 2001). The end-stopped motion selective cells can respond to corners and other two-dimensional features such as the intersections of superimposed gratings (e.g, Movshon & Newsome, 1996) producing pattern, rather than component directional responses. These responses may disambiguate an object's motion when the 2D features are intrinsic to the object (Shimojo et al., 1989). However, when they are extrinsic terminators, such as the points of intersection between the grid lines and the target contours in the furrow stimulus—they do not belong to the object and are instead accidents of occlusion that should be ignored—but are not. Indeed, the MT neurons recorded by Pack et al. (2004) did responded to terminators that were intrinsic to a moving object more than to those that were extrinsic, accidents of occlusion. However, Pack et al. (2004) were recording mostly from receptive fields in central vision where the furrow illusion is eliminated. Therefore one plausible explanation for the furrow illusion is that in the periphery, the parsing of motion signals arising from intrinsic versus extrinsic features does not occur. The two motion signals are then simply averaged to produce an illusory direction for the target (Shapiro et al., 2010; Kwon, Tadin, & Knill, 2015). We are currently exploring other stimuli to test this assumption of the breakdown of parsing in the periphery and for brief presentations as well (less than 60 ms, Pack & Born, 2001).

Interestingly, models of first-order motion energy (e.g., Adelson & Bergen, 1985; Battaje, Brock, & Rolfs, 2023) represent only the responses of typical direction selective units. The responses of end-stopped motion cells that we and others propose to explain the furrow illusion are not yet incorporated into these models. For example, the Motion Perception Tool (Battaje, et al., 2023) does not show any response to the terminators moving laterally across the furrow target.

In conclusion, although we the authors pride ourselves on our work with the furrow illusion, we did not originate it. Randolph Blake (et al.) got there first. The focus of our paper here arose from a small piece of Randolph Blake's research program unrelated to his main areas of binocular rivalry, biological motion, and blackboard sounds. Rather than being a fringe topic, though, his original furrow finding highlights the diversity of Randolph's interests, his extraordinary observation skills, and his ability to probe deeply into issues of brain mechanisms. We add our thanks to that of the others in this special issue and to that of the whole field of vision sciences for Randolph's many critical and long lasting contributions.
